# Innovative Drug Modalities for the Treatment of Advanced Prostate Cancer

**DOI:** 10.3390/diseases12050087

**Published:** 2024-05-02

**Authors:** Maurizio Capuozzo, Mariachiara Santorsola, Monica Ianniello, Francesco Ferrara, Andrea Zovi, Nadia Petrillo, Rosa Castiello, Maria Rosaria Fantuz, Alessandro Ottaiano, Giovanni Savarese

**Affiliations:** 1 Coordinamento Farmaceutico, ASL-Naples-3, 80056 Ercolano, Italy; m.capuozzo@aslnapoli3sud.it (M.C.); f.ferrara@aslnapoli3sud.it (F.F.); 2 Istituto Nazionale Tumori di Napoli, IRCCS “G. Pascale”, Via M. Semmola, 80131 Naples, Italy; mariachiara.santorsola@istitutotumori.na.it; 3 AMES, Centro Polidiagnostico Strumentale srl, Via Padre Carmine Fico 24, 80013 Casalnuovo Di Napoli, Italy; monica.ianniello@centroames.it (M.I.); nadia.petrillo@centroames.it (N.P.); rosa.castiello@centroames.it (R.C.); mariarosaria.fantuz@centroames.it (M.R.F.); 4 Ministry of Health, Viale Giorgio Ribotta 5, 00144 Rome, Italy; zovi.andrea@gmail.com

**Keywords:** prostate cancer, androgen-deprivation therapy, immunotherapy, PARP inhibitors, precision medicine

## Abstract

Prostate cancer, a prevalent malignancy affecting the prostate gland, is a significant global health concern. Androgen-deprivation therapy (ADT) has proven effective in controlling advanced disease, with over 50% of patients surviving at the 10-year mark. However, a diverse spectrum of responses exists, and resistance to ADT may emerge over time. This underscores the need to explore innovative treatment strategies for effectively managing prostate cancer progression. Ongoing research endeavors persist in unraveling the complexity of prostate cancer and fostering the development of biologic and innovative approaches, including immunotherapies and targeted therapies. This review aims to provide a valuable synthesis of the dynamic landscape of emerging drug modalities in this context. Interestingly, the complexities posed by prostate cancer not only present a formidable challenge but also serve as a model and an opportunity for translational research and innovative therapies in the field of oncology.

## 1. Introduction

**Figure 1:**
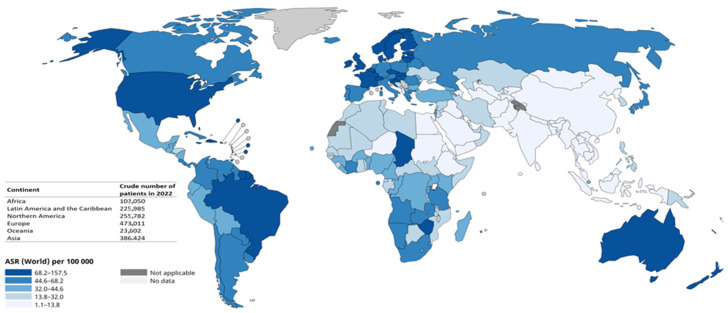
Global age-standardized rate (ASR) of prostate cancer incidence, as stated in the legend. ASR is computed by averaging age-specific rates within the population of interest and employing a standard population distribution as the weights. ASR enables comparisons of disease incidence rates across diverse populations with differing age structures.

Prostate cancer (PC) emerges as the predominant solid cancer affecting men worldwide. The estimated global incidence (rate of new PC cases occurring in 2022) is illustrated in Figure 1, revealing a total of 1,467,854 new diagnoses worldwide. Mortality, referring to the number of patients deceased from PC in the same year, exhibits heterogeneity, albeit one consistently lower compared to the incidence. The mortality-to-incidence ratio varies significantly, ranging from a very low ratio of 0.15 for Northern America to a higher ratio of 0.54 for Africa (data were extracted from the public tool of the International Agency for Research on Cancer of the World Health Organization, available at https://gco.iarc.fr/today/en, last accessed on 23April 2024).

Factors contributing to the risk of PC include confirmed factors such as advancing age (typically over 50 years old), ethnicity (AfricanAmerican men have a higher risk), and genomic alterations (germline variants of *HOXB13* and *BRCA1*/2), as well as probable factors like infections with Human Papilloma Virus-16, Neisseria gonorrhea, herpes simplex 1 and 2, Epstein–Barr virus, and Mycoplasma. Additionally, modifiable risk factors, such as obesity and dietary patterns (high intake of red and processed meats, refined grains, sweets, and high-fat dairy products,), play a role in prostate cancer risk [1,2]. Primary approaches to localized disease involve surgery and radiotherapy [3]. In cases of recurrent or metastatic disease, the standard medical treatment encompasses androgen-deprivationtherapy (ADT), inhibition of androgen signaling (ARSI), and chemotherapy. Unfortunately, over a variable period of time, patients develop castration resistance, resulting in an unfavorable prognosis [4].

The development of PC is intricately connected to the dynamic interplay of intrinsic and extrinsic elements. Persistent inflammation, commonly identified in preneoplastic prostates, is implicated in propelling the initiation and advancement of prostate carcinogenesis [5,6]. This inflammatory response can attract a variety of immune cells into the tumor microenvironment (TME), influencing the overall inflammatory milieu [7]. Cellular components within the TME exert multifaceted roles in the development and progression of PC. The TME contributes to immune remodeling and surveillance, while concurrently fostering tumor growth, metastasis, and evading immune surveillance [8]. 

This review commences with a succinct exploration of potential triggers for the initiation and progression of PC, emphasizing the potentially procarcinogenic influence of endogenous and exogenous factors during chronic inflammation development. The review also delves into the relationship between carcinogenesis-associated inflammation and the accumulation of diverse immune cells within the TME. Subsequently, the TME is thoroughly elucidated to provide profound insights into the immunobiology of PC. The subsequent sections meticulously outline innovative therapeutic approaches to PC, with a specific focus on immunotherapies and clinical insights derived from associated research. Despite advancements, the efficacy of immunotherapy, particularly immune-checkpoint blockade (ICB) therapies, in PC patients remains constrained and unsatisfactory. The inevitable resistance to ICB treatment necessitates the exploration of combination strategies to transition tumor cells from a “cold” immune state to a “hot” immune state. To conclude, we consolidate insights from recent advances, summarizing numerous agents that hold potential for combinational therapies in the treatment of PC.

## 2. Multifaceted Genesis of Prostate Cancer: Genetics, Inflammation, and Microbial Factors

**Figure 2:**
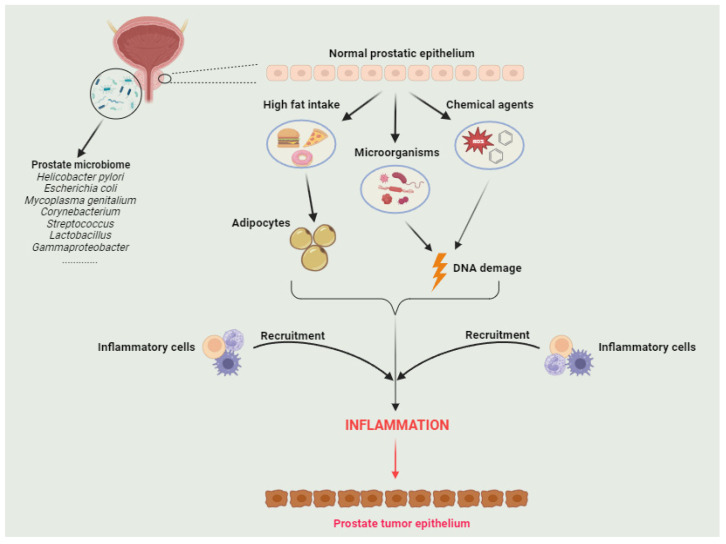
Various potential triggers, including microbial infections, chemical irritations, dietary factors, and obesity, have the capacity to induce chronic inflammation. A pathogenic alteration in the microbial species’ composition within the intraprostatic and genitourinary tracts, known as dysbiosis, may directly or indirectly contribute to an inflammatory state, thereby predisposing the epithelial barrier to compromise. Subsequently, the damage to the epithelial layer initiates an immune-system response, recruiting inflammatory cells, inducing oxidative stress, and leading to consequent DNA damage. This cascade of events triggers compensatory epithelial proliferation that promotes prostatic intraepithelial neoplasia.

The genesis of PC involves complex interactions among germline susceptibility loci, somatic gene alterations, and micro/macroenvironmental components [9]. Chronic inflammation is believed to foster the progression of various solid cancers, with well-documented instances of colon, stomach, and liver cancer [10,11,12]. While the precise mechanistic links between inflammation and PC remain undefined, chronic inflammation may play a promoting role in prostate carcinogenesis, as evidenced by the preventive and therapeutic effects of non-steroidal anti-inflammatory drugs (NSAIDs) [13]. Notably, inflammation serves as a catalyst for somatic genome and epigenome alterations, facilitated by oxidative stress and inflammatory cytokines [14]. Furthermore, inflammation may contribute to the prostate carcinogenic process by stimulating and transforming pre-malignant cells into cancer cells. In fact, inflammation in the prostate peripheral zone induces club-cell gene expression, particularly in luminal epithelial cells during proliferative inflammatory atrophy (PIA), potentially contributing to oncogenic transformation [15]. Potential factors associated with chronic inflammation implicated in PC development include microbial stressors, high dietary fat intake or obesity, chemical injury, and physical trauma [16]. Importantly, commensal microbiota have been established to colonize the gland, which is recognized as a pivotal component of the TME [17]. Recent findings have indicated the microbiome’s involvement in both the initiation and progression of prostate carcinogenesis, with potential implications for antitumor immunotherapies [18,19]. Microorganisms within the prostate may potentially stem from the urinary tract, instigating prostatic infection. This microbial infection, in turn, induces prostatic injury, compromising epithelial defenses and ultimately giving rise to chronic, persistent inflammation [20] (Figure 2). 

Another possible source of the microbiome is the gastrointestinal (GI) microbiome. Emerging evidence suggests that metabolites and androgens produced by the GI microbiome may contribute to the development of PC [21]. A comprehensive understanding of the intricate relationship between potential microbiota-associated chronic inflammation is deemed crucial for strategies aimed at preventing PC. Another notable putative factor in the development of PC is the elevated intake of dietary fats and obesity. Multiple mechanisms suggest that adipocytes surrounding the prostate gland may release chemokines or inflammatory cytokines, thereby promoting the progression and migration of PC [22]. This phenomenon could elucidate the heightened risk association between obesity and PC, offering potential therapeutic targets. Numerous studies have established a robust correlation between a high-fat Western diet and the amplified growth and metastasis of PC [23,24,25]. An intriguing recent study conducted by Labbé et al. demonstrated that a high-fat diet enhances the oncogenic MYC transcriptional signature through histone methylation at the promoter regions of MYC-targeted genes, resulting in an increased tumor burden in a murine PC model [26].

## 3. Epidemiology and Molecular Pathology

Approximately 15% of PCs are believed to have a hereditary basis [27], resulting from genetic mutations with autosomal dominant inheritance and featuring an early onset [28]. Moreover, it has been established that the incidence of PC in African American men is two to three times higher than that in European and Asian men, respectively [29,30]. This difference may be influenced by various lifestyle factors, such as diet and obesity, along with variations in screening patterns within distinct ethnic/racial communities [31]. The genetic etiology displays notable variations across diverse populations. Notably, certain single-nucleotide polymorphisms previously identified in white or Asian populations were not identified in individuals of Afro-American descent. In line with these observations, subsequent genome-wide association studies (GWAS) failed to reproduce a significant portion of the previously reported loci identified in European or Asian populations [27]. In particular, there is an increased frequency of several BRCA1/2 variants in African American patients compared to Caucasian Americans (4.6% vs. 1.6%, respectively) [32]. In a truly intriguing recent study, Rebbeck et al. demonstrated that susceptibility loci for hereditary PC were found on all chromosomes except 15, 16, 21, and 23 [33]. However, at present, the genes consistently associated with hereditary PC susceptibility have been primarily documented in the guidelines of the National Comprehensive Cancer Network (NCCN) [34]. These genes include Lynch syndrome-associated genes (MLH1, MSH2, MSH6, and PMS2) and genes involved in homologous recombination (BRCA1/2, ATM, PALB2, and CHEK2). In Table 1, we have reported additional genes, along with their respective mutation frequencies, identified in specific studies found in the current scientific literature [35,36,37,38].

Additional hereditary alterations within genes linked to DNA repair have been detected in studies on hereditary PC, including RAD51C, RAD51D, and TP53 genes. Regrettably, there are, presently, limited data available regarding the presence of such associations. Consequently, further investigations incorporating an expanded array of genes are warranted to substantiate the identification of these and other suggested candidates as susceptibility genes for hereditary PC [39].

## 4. Standard Treatment of Prostate Cancer

The inhibition of the androgen receptor (AR) stands as the primary approach in managing metastatic hormone-sensitive prostate cancer (mHSPC), as substantiated by seminal studies from 1941. These pivotal experiments demonstrated the androgen-driven and androgen-dependent nature of PC, underscored by its responsiveness to testosterone deprivation [40]. Androgen signaling plays a pivotal role in propelling the growth and survival of PC [41]. Initially introduced through surgical castration (bilateral orchiectomy) and the subsequent use of diethylstilbestrol, this therapeutic strategy evolved with the development of luteinizing hormone-releasing hormone (LHRH) agonists and antagonists, built upon the understanding of hypothalamic–pituitary control of gonadal testosterone production [40,41].

The concept of combining antiandrogens with androgen-deprivation therapy (ADT) or achieving complete androgen blockade emerged from the hypothesis that it could eliminate the activity of testicular and adrenal androgens [42]. Early-generation AR inhibitors, like flutamide, bicalutamide, nilutamide, and cyproterone acetate, are typically not employed as monotherapy. Instead, they are frequently combined with testosterone suppression (TS), referred to as combined ADT, to prevent flare responses resulting from the initial agonistic (positive feedback) effects of LHRH-agonist therapy. While an individual patient data (IPD) meta-analysis involving 8275 men from 27 randomized trials compared TS alone with combined ADT, revealing improved 5-year overall survival (OS) with nonsteroidal antiandrogens (absolute benefit 3%; two-sided *p* =0.005) and potential worsening with cyproterone acetate (absolute reduction 3%; two-sided *p* = 0.04), these findings have become foundational for considering combined ADT, incorporating weak, early-generation AR inhibitors as a potential control arm in clinical trials for mHSPC [43]. However, real-world clinical practices continue to exhibit significant heterogeneity.

From the early 1940s until 2015, TS alone, either with or without an AR inhibitor, constituted a conventional therapeutic approach for mHSPC prior to the emergence of castration resistance. In 2004, pivotal trials, TAX 327 and SWOG9916, unveiled a noteworthy enhancement in OS among men with mCRPC undergoing ADT combined with docetaxel/prednisone compared to ADT paired with mitoxantrone/prednisone [44,45]. These outcomes triggered an immediate transformation in the treatment landscape for mCRPC.

The integration of hormonal therapy with cytotoxic therapy was not only influenced by clinical trial results but also rooted in a robust scientific rationale. Advanced and resistant prostate cancer exhibits diverse clonal populations, both within and between metastases. These populations may be driven differentially by androgen-receptor-dependent and non-AR-dependent mechanisms, underlining the complexity of the disease. This insight further supports the rationale behind combining hormonal and cytotoxic therapies for an effective and comprehensive approach to address the heterogeneous nature of advanced PC [46].

It is important to highlight that not every patient is deemed suitable for docetaxel, frequently due to the presence of comorbid conditions. Additionally, radiation therapy directed at the prostate presents an OS advantage, coupled with a more favorable adverse event profile when compared to docetaxel, particularly in men with synchronous, low-volume mHSPC [47].

Based on the data available in the literature, prostate radiotherapy is an established standard for synchronous metastatic prostate cancer with a low burden/volume. However, questions persist regarding its role in combination with systemic therapy [48,49,50].

Nonetheless, the extensive literature findings substantiate the incorporation of an AR inhibitor for individuals commencing ADT alongside docetaxel, particularly in cases of synchronous high-volume metastatic ailment [51]. Additional follow-up assessments may contribute to a more lucid comprehension of the therapeutic role played by ADT combined with docetaxel in diverse clinical subcategories. Specifically, the advantages derived from the addition of docetaxel to the framework of ADT plus AR inhibitors remain undisclosed; to the best of our knowledge, there is a dearth of randomized trials reporting the outcomes of patients subjected to ADT along with AR inhibitors, with or without docetaxel. Nevertheless, an investigatory analysis of a notably intriguing investigation (ENZAMET) underscores the potential efficacy of this strategy in high-risk subgroups deliberately chosen for docetaxel treatment, showcasing inferior prostate cancer-specific survival rates [52].

## 5. Immunotherapy

In the contemporary scientific landscape, the horizon of therapeutic possibilities has expanded significantly with the advent of immunotherapies that have emerged as formidable contenders in the battle against diverse solid tumors. Notable among these are non-small-cell lung cancer, cholangiocarcinoma, triple-negative breast cancer, and melanoma [53,54,55,56], where the beacon of hope resides in the profound potential of cancer immunotherapy. The overarching objective is to instigate a robust immune response directed against tumor cells. Within the enigmatic realm of PC, tantalizing glimpses of therapeutic efficacy have materialized through the intricate tapestry of immunotherapy strategies. These encompass innovative approaches rooted in vaccination, precision modifications targeting immune cells, and interventions grounded in immune-checkpoint blockade. As we embark on this scientific journey, we unravel the multifaceted intrigue surrounding these immunotherapeutic strategies, seeking to decode their promise in the nuanced context of PC therapeutics.

**Figure 3:**
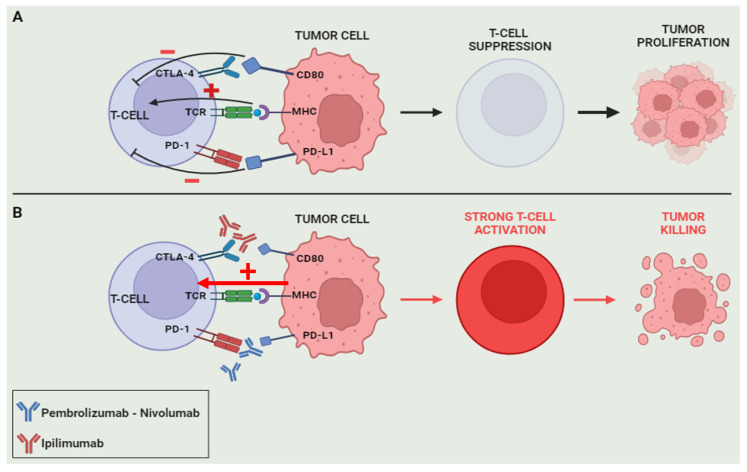
Mechanisms of immunotherapy. Tumor cells have the ability to suppress the immune response by establishing a connection between CD80/PD-L1 expressed on the tumor cell and CTLA-4/PD-1 receptors expressed on Tcells. This interaction initiates an inhibitory signal directed at Tcells, leading to the unimpeded proliferation of tumor cells (**A**). In the presence of CTLA-4/PD-1 checkpoint inhibitors, this interaction is blocked, resulting in robust T-cell activation and empowering them to effectively eliminate tumor cells (**B**).

Checkpoint proteins, intrinsic self-recognition molecules, play a crucial role in dampening the immune response to mitigate potential tissue damage in response to inflammatory stimuli [57]. While groundbreaking in various cancer treatments, immune-checkpoint inhibitors have encountered limited success in addressing PC, partly due to their immunologically inert nature. CTLA-4, a T-cell-expressed immune-checkpoint receptor homologous to the cluster of differentiation (CD) 28, demonstrates potential when inhibited, as exemplified by agents like ipilimumab, fostering increased T-cell activation and infiltration into tumors [58]. Conversely, PD-1, a co-signaling receptor within the B7/CD28 family, found on activated Tcells, Bcells, natural killer cells, and exhausted Tcells, encounters its ligand PD-L1 extensively expressed on some tumor cells as an evasion mechanism against the host’s immune system. PD-1, upon binding with PD-L1, dampens T-cell receptor signaling, diminishing T-cell activity and effector functions in peripheral tissues [59]. Disrupting this interaction reinstates T-cell activity in the periphery. Though immune checkpoints are pivotal in safeguarding host tissue from autoimmune responses, tumors exploit these regulatory mechanisms to elude immune surveillance. By masquerading as ‘self’ through heightened PD-L1 expression on their surface, tumors disguise themselves, evading detection by the immune system [59,60] (Figure 3). 

The assessment of monoclonal antibodies targeting PD-1 and CTLA4, such as pembrolizumab and ipilimumab, has been conducted in patients, suggesting potential efficacy in those progressing onto enzalutamide. However, the results are not satisfactory, and the precise role of immune-checkpoint blockade (ICB) in PC remains uncertain. 

A comprehensive description of the trials and their outcomes is provided in Table 2 [61,62,63,64,65,66,67,68,69,70,71,72,73,74,75], where key messages can be highlighted. It is likely necessary to identify and select patients who may benefit more effectively from immunotherapy. Interestingly, inactivating mutations in the cyclin-dependent kinase CDK12 (found in up to 7% of mCRPC tumors) may be associated with responsiveness to ICB in mCRPC [66]. Currently, a phase II trial investigating ipilimumab and nivolumab in patients with tumors harboring CDK12 mutations is actively ongoing (NCT03570619) [67]. Similarly, another phase II trial explored the combination of ipilimumab and nivolumab in PC patients with AR-V7 mutations, revealing that those with mutations in DNA-repair genes exhibited more favorable biochemical and radiographic responses [72]. Additionally, ongoing research is investigating the combination of PARP inhibitors with ICB (NCT02484404), representing an innovative frontier of treatment [73]. Continued evaluation of combination therapies involving ICB in PC patients represents the optimal approach for validation, especially considering the limited efficacy observed with other ICB monotherapies.

The constrained efficacy of immunotherapy in treating PC can likely be attributed to several factors, including the presence of alternative immune-checkpoint pathways, low mutational burden in PC tumor cells, and deficiency of immune cells, particularly T cells, infiltrating the tumor microenvironment and creating a characteristic “cold” signature compared to other immunological cancers displaying a more robust “hot” signature. These factors collectively contribute to the limited benefits observed with ICB therapies in PC. Therefore, addressing this challenge requires in-depth investigations into the fundamental mechanisms, coupled with preclinical research focused on the potential of combination ICB therapies. The ultimate goal is to transition PC from a “cold” to a more responsive “hot” phenotype.

## 6. Integrating Vaccine-Based and Dendritic Cell Immunotherapies

**Figure 4:**
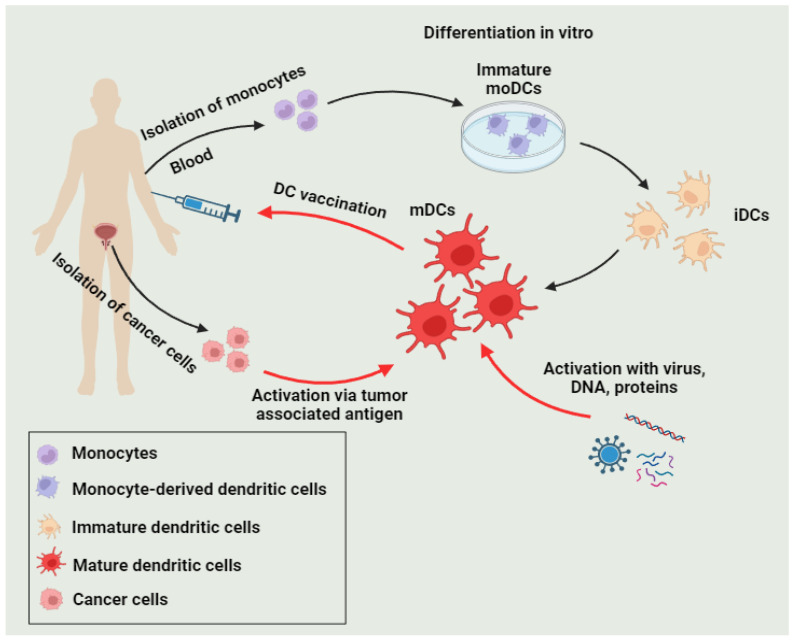
The idea behind DC vaccination involves utilizing autologous monocytes as a prevalent source for DC vaccines in clinical trials. These monocytes are subjected to differentiation and maturation in a controlled environment in vitro. Once loaded with tumor-associated antigens (or virus, DNA, proteins, peptides), the resulting DC vaccines are administered through infusion to stimulate a targeted T-cell response.

Dendritic cells (DCs) act as a crucial link between innate and adaptive immune responses, presenting tumor-associated antigens to enhance potent, antigen-specific T-cell responses against cancer cells. Harnessing these unique features, researchers have developed DC-based vaccines as a promising avenue in cancer immunotherapy. The standard protocol for preparing DC vaccines involves the isolation of monocytes from patients, followed by their culture with stimulatory cytokines, such as granulocyte–macrophage colony-stimulating factor (GC-CSF) and IL-4. This process transforms the monocytes into mature and activated DCs with enhanced antigen-presenting capabilities. Following this, DCs are infused with a varied range of cancer-related antigens, covering tumor peptides, proteins, messenger RNAs (mRNAs), cellular lysates, and, notably, apoptotic tumor cells. The diversity in choosing tumor antigens facilitates a thorough and customized strategy for addressing distinct cancer varieties. After being laden with antigens, the modified DCs are reintegrated into the patient, triggering an elevated and precise immune reaction against the tumor [76] (Figure 4).

Sipuleucel-T, an autologous cellular immunotherapy, represented a significant breakthrough as the first FDA-authorized DC-based vaccine in 2010 for treating patients with asymptomatic or minimally symptomatic mCRPC [77]. The therapy involves the utilization of autologous peripheral blood mononuclear cells, reintroduced to patients after in vitro culture. The preparation includes a recombinant fusion protein-containing prostatic acid phosphatase, prostate antigen, and GM-CSF [78]. This fusion protein activates antigen-presenting cells (APCs), facilitating antigen expansion [79]. A pivotal double-blind phase III randomized multicenter study (IMPACT) enrolled 512 patients in a 2:1 ratio to receive either Sipuleucel-T or a placebo, with OS as the primary endpoint. The experimental group displayed a 22% relative reduction in the risk of death compared to the placebo group, along with a 4.1-month increase in median survival (25.8 months vs. 21.7 months). The 36-month survival probability was 31.7% for Sipuleucel-T versus 23.0% for the placebo. The most frequent adverse events in the Sipuleucel-T group were headache and fever [80]. Currently, it remains the only FDA-approved vaccine for PC. However, concerns regarding trial outcomes and the associated high cost have hindered its broad acceptance in clinical practice [81]. Regrettably, PROSTVAC, a dendritic cell-based vaccine akin to Sipuleucel-T, failed to demonstrate an overall survival advantage in a phase III study [82]. While our comprehension of DC vaccines has expanded over the past decade, no additional DC therapy has been established to date, indicating a potential gap between fundamental research and clinical application requiring exploration. Another vaccine, GVAX, relies on genetically modified PC cells producing GM-CSF [83]. GVAX is a safe cytokine provoking an immune response in a dose-dependent manner, with patients experiencing only fever and flu-like symptoms during treatment. However, due to several unsuccessful phase III studies, further trials have been discontinued [84,85]. Recent investigations into Sipuleucel-T and PROSTVAC have shifted towards combination therapies. A phase II study pairing Sipuleucel-T with radium-223 for mCRPC patients demonstrated a synergistic effect. Specifically, the combination led to significant PSA decline, prolonged PFS (39 vs. 12 weeks; hazard ratio [HR], 0.32; 95% confidence interval [CI], 0.14–0.76), and improved OS (not reached vs. 2.6 years; HR, 0.32; 95% CI, 0.08–1.23) [86]. However, another phase II trial reported no increase in time to progression or OS when combining Sipuleucel-T with stereotactic ablative radiotherapy for mCRPC patients compared to the original IMPACT clinical trial [87]. PROSTVAC was also explored in combination with ADT for mCRPC patients [88]. The characteristics of the ongoing studies investigating the combination of these vaccines with the other targeted agents in PC patients are listed in Table 3.

## 7. Targeted Therapy in Prostate Cancer

The integration of molecular diagnostics has deepened our comprehension of potential therapeutic pathways in PC, particularly among mCRPC patients. Homologous recombination regulation by BRCA 1 or 2 can be compromised through germline or sporadic alterations. Cancers with BRCA deficiencies exhibit explicit susceptibility to Poly (ADP-ribose) polymerase (PARP) inhibitors [88,89]. Oral targeted treatments like olaparib and rucaparib have gained approval for application in mCRPC patients. PARP is a crucial protein in single-stranded DNA break repair. Inhibition of PARP results in the accumulation of double-stranded breaks. In individuals lacking double-stranded break-repair mechanisms, this induces synthetic lethality and cell death [90]. The mutation status of BRCA1/2 can significantly impact the choice of treatment. BRCA loss leads to homologous recombination deficiency, making cells responsive to both platinum chemotherapy and inhibitors targeting the DNA repair enzyme PARP. In mCRPC, around 15–20% of patients exhibit genetic alterations in homologous recombination repair (HRR) genes, resulting in the authorization of PARP inhibitors for this specific patient group [91]. The PROfound investigation contrasted olaparib with abiraterone acetate or enzalutamide in individuals exhibiting deficiencies in HRR after undergoing treatment with a novel hormonal agent for CRPC. Among participants in the Olaparib group, BRCA1 alterations were identified in 8 out of 256 patients (3%), while in the control group, 5 out of 131 patients (4%) exhibited BRCA1 alterations. BRCA2 alterations were present in 32% of the Olaparib group and 36% of the control group. In individuals with at least one alteration in BRCA1, BRCA2, or ATM, Olaparib demonstrated improved rPFS (7.39 vs. 3.55 months, HR = 0.34; 95% CI: 0.25–0.47; *p*< 0.0001) and OS (18.5 months vs. 15.1 months, hazard ratio for death = 0.64; 95% CI, 0.43 to 0.97; *p* = 0.02). The interim analysis for the entire population revealed a median OS of 17.5 months (Olaparib group) and 14.3 months (control group) (hazard ratio for death = 0.67; 95% CI, 0.49 to 0.93). Notably, patients with BRCA alterations experienced the greatest benefit, prompting a restricted label in Europe. However, the sensitivity of other HRR alterations to PARP inhibitors is apparent, although subgroups are limited, and their predictive strength remains uncertain [92]. Rucaparib, conversely, holds approval from the FDA for individuals with BRCA1 or BRCA2 alterations who have experienced progression on ARAT treatment and taxane chemotherapy [93]. The standard evaluation of germline or somatic mutations in BRCA1 and BRCA2 from either tumor or liquid biopsy is now a routine procedure, serving as a predictive biomarker for the administration of PARP inhibitors in second-line treatment for mCRPC [94,95]. Ongoing investigations involve other PARP inhibitors, including talazoparib and niraparib [96,97,98]. Intriguingly, a class effect appears to be present, with PARP inhibitors exhibiting heightened efficacy in patients with BRCA2 mutations in comparison to BRCA1 [99]. A meta-analysis focusing on PARP inhibitors in mCRPC highlighted the effectiveness of BRCA mutations and HRR mutations as predictive biomarkers for the response to PARP inhibitors in this patient population [100]. Additionally, alterations of the phosphatidylinositol-3-kinase (PI3K)/AKT signaling pathway are common in PC. Several studies have investigated PI3K and AKT inhibitors for treating PC patients. For instance, ipatasertib, an AKT inhibitor, is currently being studied in combination with abiraterone. In a multicenter, randomized, double-blind, phase 3 trial (IPATential150 study), the combination of pimasertib with abiraterone and prednisolone demonstrated improved rPFS (16.5 months vs. 18.5 months, HR 0.77, CI 0.61–0.98, *p* = 0.034) and ORR compared to abiraterone and prednisolone with placebo in patients with phosphatase and tensin homolog (PTEN) loss mutations [101]. Another AKT inhibitor, capivasertib, when combined with docetaxel, showed prolonged OS in a phase II study, prompting a further investigation to identify patients who could benefit from this combination [102]. Additionally, capivasertib, in combination with enzalutamide, is undergoing phase I trials [103]. AKT inhibitors exhibit promise in mCRPC treatment, particularly in cases of acquired resistance to PARP-inhibitor monotherapy, and are under investigation in combination with PARP inhibitors. A novel therapeutic approach has emerged with systemic prostate-specific membrane antigen (PSMA)-targeted radio-ligand therapy (RLT) in mCRPC. Lutetium-177-PSMA-617 (LU-PSMA) is a small molecule that specifically binds to PSMA, allowing ß particle therapy for adjacent tumor cells. A positive diagnostic 68-Gallium PSMA PET scan is a prerequisite to selecting eligible patients for this molecular therapy [104]. The VISION trial evaluated LU-PSMA in previously treated mCRPC patients ineligible for chemotherapy. LU-PSMA demonstrated significant benefits in rPFS (8.7 vs. 3.4 months, *p*< 0.001; HR = 0.40; 99.2% CI: 0.29–0.57) and OS (15.3 vs. 11.3 months, *p*< 0.001; HR = 0.62; 95% CI: 0.5–0.74) compared to the standard of care (including hormonal therapy, denosumab, bisphosphonates, radiation therapy, or glucocorticoids) [105]. Both BRCA1/2 mutations and PSMA-positivity served as predictive markers for treatment benefit.

## 8. Antibody–Drug Conjugates in PC Treatment

Tumor cells of various malignancies, including prostate cancer (PC), often exhibit elevated expression of specific antigens, offering opportunities for targeted therapeutic interventions. Within this context, antibody–drug conjugates (ADCs) have emerged as an innovative pharmacological approach [106]. By utilizing monoclonal antibodies (mAbs) precisely targeted to unique tumor antigens, ADCs are engineered with cytotoxic payloads linked to the antibody component. This novel strategy not only broadens therapeutic windows but also reduces the risk of off-target toxicities, marking a significant advancement in precision oncology [107]. 

**Figure 5:**
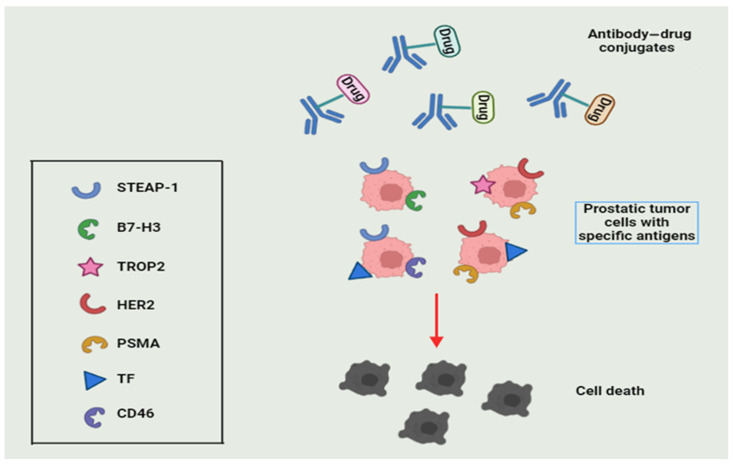
The antibody–drug conjugate selectively attaches to prostate tumor cells through antigen recognition. Subsequently, upon internalization, the cytotoxic drug is released, leading to cell death.

Although ADCs have demonstrated considerable clinical efficacy in certain cancer types, notably breast cancer, research into their application in PC treatment is steadily expanding. Currently, researchers are particularly interested in targeting six-transmembrane epithelial antigen of the prostate 1 (STEAP-1), B7 homolog 3 protein (B7-H3), trophoblast antigen 2 (TROP2), human epidermal growth factor receptor 2 (HER2), folate-hydrolase 1 (PSMA), tissue factor (TF), and cluster of differentiation 46 (CD46) with ADCs [108,109] (Figure 5). Ongoing technological advancements are continuously improving target specificity, linker design, and payload selection, facilitating the development of promising next-generation ADCs [110]. Conjugate synthesis benefits from progress in protein engineering and biochemistry. Moreover, these novel ADCs may incorporate bispecific monoclonal antibodies, enabling simultaneous targeting of multiple antigens [111]. Among the challenges to overcome, one significant obstacle is the potential resistance of tumor cells, primarily attributed to structural changes in target antigens or decreased antigen expression. Overcoming this important mechanism of resistance is crucial to ensure the success of ADCs in the therapeutic landscape of PC and other solid tumors. 

Although we are in the early stages of clinical exploration of these agents for PC, the available studies, including some basket trials not oriented toward specific diseases, demonstrate a manageable safety profile and encourage antitumor activity in heavily pretreated PC patients [112,113,114,115,116,117,118]. These breakthroughs offer the prospect of enhancing the evaluation of ADCs, thereby considering innovative combinations in the near future to improve clinical outcomes.

## 9. Future Challenges

PC immunotherapy faces numerous challenges, including the delicate balance between the effectiveness and toxicity of the treatment, optimal timing for sequential administration, the necessity for personalized dosing regimens due to tumor heterogeneity, the absence of suitable biomarkers for evaluating efficacy, and a limited understanding of drug-resistance mechanisms. These challenges require dedicated attention in future research endeavors. Given the substantial variations across immunotherapy studies, there is a notable absence of direct evidence supporting the comparison of treatment effects from diverse regimens. Future investigations should prioritize conducting more large-scale controlled trials to address this gap. The realm of PC immunotherapy holds significant promise. Ongoing research has uncovered new tumor-specific antigens, expanding the array of potential targets for immunotherapeutic interventions. Advances in high-throughput sequencing technology and liquid biopsy techniques have facilitated a better understanding of PC’s tumor heterogeneity, thereby enhancing precision in patient treatment. The continual exploration of drug combinations contributes to elucidating the mechanisms of drug interactions. 

An especially intriguing frontier, still in its preclinical stages, is the utilization of nucleic acids including microRNAs (miRNAs) and small interfering RNAs (siRNAs) as innovative therapies for prostate cancer [119,120,121]. Unfortunately, none of these agents have yet entered a clinical validation pathway. The purpose of this discussion is not to delve exhaustively into delivery systems or specific inhibited pathways. However, it is noteworthy that these small non-coding RNAs play pivotal roles in post-transcriptional gene-expression regulation and are implicated in various cellular processes, encompassing proliferation, apoptosis, and differentiation. In castration-resistant prostate cancer (CRPC), dysregulated miRNA expression profiles have been observed, contributing to tumor progression and therapeutic resistance. One of the primary advantages of employing miRNAs and siRNAs as therapeutic agents lies in their capacity to concurrently target multiple genes, thereby influencing various pathways involved in cancer progression. This multifaceted mode of action renders them particularly appealing for CRPC treatment, which is characterized by intricate molecular alterations. Similarly, these agents can be tailored to specifically target critical pathways implicated in metastatic CRPC (mCRPC) pathogenesis, such as those associated with cell cycle regulation, apoptosis, and angiogenesis. Through selective gene silencing linked to these pathways, siRNA therapy holds the potential to exert potent anti-tumor effects and overcome resistance mechanisms that contribute to disease advancement. Preclinical investigations have illustrated the potential of miRNA- and siRNA-based therapies in CRPC by reinstating the expression of tumor-suppressor genes or inhibiting oncogenes [119,120,121].

Unraveling the intricate mechanisms underlying both primary and acquired resistance remains a paramount focus for advancing second-generation therapeutic strategies. Investigating molecular profiles of tumors and systemic immune parameters, both before and after progression on immunotherapies, can shed light on aberrant pathways that foster immunosuppression or immune evasion. Such insights have the potential to reveal actionable targets, paving the way for innovative approaches to counter resistance. In addition to exploring combinations and deciphering resistance mechanisms, a critical stride lies in identifying robust predictive biomarkers to enhance the selection of potential responders to immunotherapy. Leveraging technologies, such as multiplex immunohistochemistry, tracking mutations through liquid biopsy, and integrating multilayered datasets through machine learning, may facilitate the prospective identification of patients most likely to derive benefits [122]. Currently, among the most prospective molecular targeted agents [123], there is a notable focus on drugs targeting DNA methylation and demethylation. In preclinical studies and clinical trials for treating mCRPC, various agents, such as histone acetyltransferases, deacetylases, demethylases, methyltransferases, and DNA methyltransferase inhibitors, demonstrate anti-tumoral effects primarily through gene-expression reprogramming [124,125,126]. A cutting-edge approach in molecular cancer targeting, including PC, involves nanomedicine-based strategies. These strategies aim to enhance drug delivery, improve treatment efficacy, and minimize side effects. Nanoparticles with diverse formulations, such as liposomes, polymeric nanoparticles, and inorganic nanoparticles, can be tailored to encapsulate molecular targeted drugs. Through conjugation with antibodies recognizing tumor-associated markers like PSMA, these nanoparticles selectively accumulate in prostate tumors, enhancing targeting and internalization. Notably, nanoparticles can deliver therapeutic genes to both PC cells and cells of the TME. As these innovative therapeutic agents have entered clinical practice relatively recently, data regarding their long-term effects remain unavailable. Therefore, it is crucial to meticulously evaluate the impact of these therapies on the health status of long-surviving patients, who often present with other comorbidities.

## 10. Conclusions and Perspectives

Our review emphasizes a significant challenge in managing advanced hormone-resistant PC. Despite considerable progress in biological therapies, there remains a deficiency in achieving satisfactory outcomes regarding both objective response rates and 5-year survival. This observation underscores the pressing need for a more profound comprehension of the intricate genetics and molecular dynamics underlying this pathology, a realm that remains elusive in current scientific understanding. To address this knowledge gap, a strategic shift toward experimental research is imperative. Returning “to the bench” becomes not merely a suggestion but a pivotal necessity. This approach offers an opportunity to explore novel dimensions beyond the scope of conventional therapeutic targets. In particular, the investigation of alternative immune-checkpoint blockers and the exploration of new molecular pathways emerge as promising avenues. By delving into these uncharted territories, we can potentially uncover unprecedented insights that may pave the way for more effective and tailored therapeutic interventions. Moreover, the complexity of advanced PC demands a comprehensive and integrated approach. Collaborative efforts across disciplines, such as genomics, proteomics, and systems biology, should be harnessed to create a holistic understanding of the disease. This collaborative framework can enhance our ability to identify biomarkers, decipher intricate signaling networks, and ultimately formulate more precise and personalized treatment strategies.

## Figures and Tables

**Table 1:** Frequency of mutations found in genes related to hereditary prostate cancer.

Acronym	Full Name	Frequency of Mutation
*ATM*	ATM serine/threonine kinase	1.6–2.7%
*BRCA1*	Breast cancer gene 1	0.9–1.25%
*BRCA2*	Breast cancer gene 2	1.2–5.3%
*BRP1*	BRCA1 interacting protein C-terminal helicase 1	0.1–0.2%
*CHECK2*	Checkpoint kinase 2	1.8–2.8%
*HOXB13*	Homeobox B13	0.6–6.25%
*MMR*	Mismatch repair	0.7–1.7%
*NBS1*	Nijmegen breakage syndrome 1	0.1–0.2%
*PALB2*	Partner and localizer of BRCA2	0.4–0.5%

**Table 2:** Overview of major clinical studies on novel immunotherapeutic agents in advanced castration-resistant prostate cancer: monotherapy and combination approaches.

Study, Ref	NCT	Phase	No. of Patients	Study Arms	Primary Endpoint	Results
KEYNOTE-028	NCT02054806	I	477	Pembrolizumab	ORR	ORR of approximately 17% (95% CI, 5.0–38.8%)
KENIOTE-199	NCT02787005	II	388	Pembrolizumab (3 patient cohorts,1docetaxel pretreated pts, 2 one previous targeted endocrine therapies, 3 docetaxel, and more than one previous targeted endocrine therapies)	ORR	Cohort 1: ORR of 5% (95% CI, 2–11%) and a median OS of 9.5 months. Cohort 2 showed a 3% ORR (95% CI, <1%–11%) and a median OS of 7.9 months. Cohort 3 presented a median OS of 14.1 months
CA184-095	NCT01057810	III	837	Ipilimumab Placebo	OS	High dose did not reach OS over placebo (28.7 months vs. 29.7 months; HR = 1.11, 95% CI 26.1–34.2 months, *p* = 0.3667).
PCD4989g	NCT01375842	I	661	Atezolizumab (dose escalation)	DLTs ORR	Good safety profile—ORR of 12 months survival of 55.6% and six-month progression-free survival rate of 26.7%
IMPACT	NCT03570619	II	56	Nivolumab Ipilimumab	ORR	No results posted (still active)
CA184-043	NCT00861614	III	988	Ipilimumab Placebo	OS	High dose did not reach OS over placebo (11.04 months vs. 10.2 months; 95% CI 9.46 to 12.48 vs 8.38 to 11.17 months).
CheckMate 650		II	90	Nivolumab plus ipilimumab pre-chemotherapy and post-chemotherapy	ORR	ORR of 25% and 10%, and median overall survival was 19.0 and 15.2months in pre- and post-chemotherapy cohorts, respectively.
IRB00011025	NCT02312557	II	58	Pembrolizumab + Enzalutamide	PSA (Response)	The results indicated a decline in PSA for 18% of patients, and 25% achieved an objective response
KENYOTE-641	NCT03834493	III	1244	Pembrolizumab + Enzalutamide Enzalutamide + placebo	OS rPFS	OS Median (95% CI) month: 24,7 (22.0–26.8) in arm1 and 27.3 (24.5–30.1) in arm2 rPFS Median (95% CI) month: 10.4 (8.4–12.5) in arm 1 and 9.0 (8.3–11.5) in arm 2
STARVE-PC	NCT02601014	II	32	Nivolumab + ipilimumab Enzalutamide + nivolumab + ipilimumab	PSA	Number of participants with greater than 50% decline in PSA from start of treatment, sustained for ≥4 weeks: 2 (13.3%) in arm 1 and 0 (0.0%) in arm 2
Unnamed	NCT02484404	I/II	384	Durvalumab + cediranib Durvalumab + olaparib Durvalumab + cediranib + olaparib	ORR	No results posted (still active)
INSPIRE (ClinicalTrials.gov)	NCT04717154	II	75	Ipilimumab + nivolumab	DCR	No results posted (still active)
CA209-935 (ClinicalTrials.gov)	NCT03061539	II	380	Ipilimumab + nivolumab	CRR	No results posted (still active)

CI: confidence interval; CRR: clinical response rate; DLTs: dose-limiting toxicities; DCR: disease control rate; HR: hazard ratio; ORR: overall response rate; OS: overall survival; PC: prostate cancer; PSA: prostate-specific antigen; rPFS: radiographic progression-free survival.

**Table 3:** Ongoing clinical trials investigating the integration of vaccine-based and dendritic cell immunotherapies in advanced PC patients (Source: ClinicalTrials.gov, accessed on 30 December 2023).

NCT Number	Status	Phase	Interventions	Key Outcome Measures
NCT01197625	Active, not recruiting	I/II	DC-vaccine	Time to treatment failure defined by two different measurements of PSA levels >0.5 µg/L with minimum of 4 weeks interval in patients receiving treatment; safety and toxicity of vaccination. Evaluation of immunological response.
NCT05010200	Recruiting	I	PGV-001, Poly-ICLC vs. PGV-001, Poly-ICLC, CDX-301	Number of adverse events; change in immune cell subsets; change in the frequency of vaccine epitope-specific T lymphocyte populations; adiographic free survival.
NCT04701021	Active, not recruiting	I	TENDU	Assessment of safety and tolerability of TENDU vaccine; assessment of Immunological response; assessment of anti-tetanus protein and anti-MTTE titers; assessment of anti-tumor activity.
NCT02649855	Active, not recruiting	II	ADT, PROSTVAC-V, PROSTVAC-F, Docetaxel	Antigen spreading; antigen-specific T-cell immune composite response scores between all arms at 39 weeks and 1 year; number of participants with T-cell response to PSA.
NCT03315871	Active, not recruiting	II	PROSTVAC-V, PROSTVAC-F, MSB0011359C, CV301	To determine if combination immunotherapy can result in 30% decline in PSA; slope of the PSA change over time; fraction of subjects with grade 3 and grade 4 adverse events.
NCT04090528	Recruiting	II	pTVG-HP, pTVG-AR, Pembrolizumab	PFS; Overall objective response rate; prostate-specific antigen response rate; median radiographic progression-free survival; median duration of PSA and objective response; OS; antigen-specific Th1 immune response; toxicity rates.
NCT03600350	Active, not recruiting	II	Nivolumab, pTVG-HP, GM-CSF	Number of Participants who experienced adverse events grade 3 or higher; PSA; CRR; metastasis-free survival rate; median radiographic progression-free survival; number of participants receiving GM-CSF as an adjuvant after week 4.
NCT05104515 *	Recruiting	I	OVM-200	Occurrence and intensity of adverse events; immune response to OVM 200, as measured by ELISpot for T cell responses and ELISA for antibody responses; in prostate cancer patients, tumor markers PSA.
NCT04382898	Active, not recruiting	I/II	BNT112 vs. BNT112, cemiplimab	DLTs; TEAEs; ORR; change PSA levels; PSADT; and tumor response post-treatment compared to baseline.
NCT02933255	Active, not recruiting	I/II	PROSTVAC-V/F, Nivolumab	Safety; evaluate changes in T-cell infiltration in the tumor after neoadjuvant treatment; evaluate changes in PDL-1 expression; evaluate changes in immune cell subsets in the periphery;
NCT04989946	Recruiting	I/II	Degarelix vs. Degarelix, Nivolumab, pTVG-AR vs. Degarelix, pTVG-AR	pCR; MRD; incidence of adverse events; toxicity rates; progression-free survival (PSA) at 1-year; RCB.
NCT04114825	Active, not recruiting	II	RV001V vs. Placebo	Time to PSA progression; safety by frequency and severity of adverse events; time to initiation of a subsequent antineoplastic therapy; proportion of patients showing a PSA response from baseline; DFS.
NCT05533203	Recruiting	I	PRODENCEL	Incidence of treatment-emergent adverse events during induction immunization; incidence of treatment-emergent adverse events during booster immunization.
NCT05751941	Recruiting	II	Abiraterone, Enzalutamide, Apalutamide, Sipuleucel-T vs. Sipuleucel-T	Cumulative APC Activation; time to PSA progression; radiographic PFS; IgG responses.
NCT05806814	Recruiting	I	Sipuleucel-T	Proportion of patients completing 3 doses of Sipuleucel-T immunotherapy; proportion of subjects who have detectable elevated IgG level and/or T-cell proliferation from baseline to the follow-up of extended course of Sipuleucel-T immunotherapy.
NCT03686683	Active, not recruiting	III	Sipuleucel-T	To assess the efficacy of Sipuleucel-T in reducing histopathologic reclassification to a higher Gleason grade in prostate cancer subjects on active surveillance.

ADT: androgen-deprivation therapy; APC: antigen-presenting cell; BNT112: mRNA-based cancer vaccine that encodes cancer-selective antigens; CDX-301: soluble recombinant human protein to work on stem cell; CRR: complete response rate; CV301: recombinant vaccinia virus vaccine of the genus Avipoxvirus; DC: dendritic cell; DFS: disease-free survival; DLTs: dose-limiting toxicities; GM-CSF: granulocyte-macrophage colony-stimulating factor; MRD: minimal residual disease; MSB0011359C: fully human bifunctional fusion protein that combines IgG1 anti-PD-L1 and TGFbetaRIIas a monoclonal antibody; MTTE: minimal tetanus toxin epitope; ORR: objective response rate; OS: overall survival; OVM-200: vaccines based on mRNA or viral particles developed using OVM’s recombinant overlapping peptides; pCR: pathological complete response rate; PFS: progression free survival; PGV-001: personalized genomic peptide vaccine; Poly-ICLC: immune modulator; PRODENCEL: autologous dendritic cell therapeutic tumor vaccine; PROSTVAC-F: recombinant fowlpox virus vector vaccine of the genus Avipoxvirus; PROSTVAC-V: recombinant vaccinia virus vector vaccine of the genus Orthopoxvirus; PSA: prostate-specific antigen; PSADT: change in PSA doubling time; pTVG-AR: plasmid DNA; pTVG-HP: plasmid DNA; RCB: residual cancer burden. RV001V: peptide cancer vaccine; TEAEs: treatment-emergent adverse events; TENDU: therapeutic peptide conjugate vaccine.*This study enrolls also ovarian cancer and non-small-cell lung cancer patients.

## Data Availability

No new data were generated in relation to the current study.
